# ELABELA-APJ Axis Enhances Mesenchymal Stem Cell Proliferation and Migration via the METTL3/PI3K/AKT Pathway

**DOI:** 10.32607/actanaturae.17863

**Published:** 2024

**Authors:** D. Xu, J. Fu, X. Liu, Y. Hong, X. Chen, S. Li, J. Hou, K. Zhang, C. Zhou, C. Zeng, G. Zheng, H. Wu, T. Wang

**Affiliations:** Department of Emergency, the Eighth Affiliated Hospital of Sun Yat-sen University, Shenzhen, Guangdong, 518003 China; Department of Emergency, the Sun Yat-sen Memorial Hospital of Sun Yat-sen University, Guangzhou, Guangdong, 510120 China

**Keywords:** ELABELA, METTL3, Mesenchymal stem cells, Apelin receptor, Proliferation, Migration

## Abstract

Mesenchymal stem cells (MSCs) possess a strong therapeutic potential in regenerative medicine. ELABELA (ELA) is a 32 amino acid peptide that binds to the apelin peptide jejunum receptor (APJ) to regulate cell proliferation and migration. The aim of this study was to investigate the function of ELA vis-a-vis the MSC proliferation and migration, and further explore the underlying mechanism. We demonstrated that the exogenous supplement of ELA boosts the proliferation and migration ability of MSCs, alongside improved *in vitro *cell viability. These capabilities were rendered moot upon APJ knockdown. In addition, ELA (5−20 μM) was shown to upregulate the expression of METTL3 in a concentrationdependent pattern, a capacity which was suppressed by APJ reduction, whereas the downregulation of METTL3 expression blocked the beneficial effects induced by ELA. ELA was also observed to upregulate the phosphorylation level of AKT. This ELA-induced activation of the PI3K/AKT pathway, however, is inhibited with knockdown of METTL3. Our data indicate that ELA could act as a promoter of MSC proliferation and migration *in vitro *through the APJ receptor, something which might be attributed to the activation of the METTL3/PI3K/AKT signaling pathway. Therefore, ELA is a candidate for optimizing MSC-based cell therapy, while METTL3 is a potential target for its promoting action on MSCs.

## INTRODUCTION

Mesenchymal stem cells (MSCs) have attracted significant attention in the field of tissue repair and regenerative medicine for their differentiation potential, homing capacity, and self-renewal abilities [1, 2]. Due to their ease of extraction, the not-sothorny ethical considerations associated with then, and their immunologic privilege, MSCs have become the most widely used stem cells in the regeneration of injured cells and tissues [3]. They can migrate to the sites of damage [4] and differentiate into the desired cell type [5] or contribute beneficial elements [6] such as growth factors. Nevertheless, the curative potential of MSCs remains limited for the following reasons: The insufficient number of MSCs collected from donors [7], the low chance of survival in a hostile environment [8], and the insufficient number of cells capable of migrating to damaged sites [9]. The migration ability is crucial for MSCs, because they better exert their therapeutic effects at the sites of damage [10]. Therefore, exploring ways to improve the *in vitro *expansion and migration capability of MSCs could be key in fulfilling the therapeutic potential of MSCs in cell-based therapy, opening up broader prospects in future regenerative medicine.

ELABELA (ELA, also known as Apela/Toddler) is a small peptide consisting of 32 amino acids that binds to APJ to form an essential signaling axis for modulating cellular events such as migration [11]. For example, ELA is an activator which promotes the movement of mesendodermal cells during the formation of zebrafish gastrulation [12], while ELA-APJ signaling is indispensable for angioblast migration towards the midline during vasculogenesis [13]. Of note, studies have confirmed that ELA expression is rapidly downregulated during the differentiation of human embryonic stem cells (hESCs) [14], and that this manifests itself in paracrine fashion to promote the proliferation of hESCs by accelerating cell-cycle progression. ELA can also activate the PI3K/AKT/ mTORC1 signaling cascade, which is required for cell survival [15]. The PI3K/AKT signaling pathway occupies a prominent place in all manners of cellular behavior of MSCs such as proliferation [16], migration [17], and apoptosis [18]. Hence, the question of whether ELA could affect the proliferation and migration of MSCs piqued our interest. We have previously confirmed that ELA reduces MSC apoptosis by stimulating the PI3K/AKT pathway under ischemic and hypoxic conditions [19], but the effect of ELA on the expansion and migration of MSCs remains unclear, with the regulatory mechanisms requiring further investigation, as well.

N^6^-methyladenosine (m_6_A) is a dynamic modification in eukaryotic RNAs that plays a pivotal role in the regulation of cellular processes [20]. A flurry of recent discoveries has pointed to the strong relationship between m_6_A modification and stem cell regulation [21, 22]. As the main component of the m_6_A methyltransferase complex (MTC), methyltransferase- like 3 (METTL3) has a direct influence on cell survival, differentiation potential, stem cell maintenance, and more [23]. The depletion of METTL3 was shown to promote cell differentiation and reduce cell proliferation in human hematopoietic stem/progenitor cells (HSPCs) [24]. Conditional knockout of METTL3 in MSCs induced pathological phenotypes of osteoporosis and brought about damaged bone formation, enhanced adipogenic capacity, together with incompetent osteogenic differentiation potential in [25]. Meanwhile, METTL3 participates in the regulation of the PI3K-AKT signaling pathway too. During the osteogenic differentiation process, the protein levels of METTL3 increased, whereas the knockdown of METTL3 suppressed AKT phosphorylation and decreased the osteogenic differentiation of MSCs in [26]. This evidence suggests that METTL3 is closely related to the lineage allocation of MSCs. Nevertheless, the role of ELA and the impact of its interaction with METTL3 on the proliferation and migration of MSCs are poorly understood and require further investigation.

In this study, we investigated the effects of ELA on the proliferation and migration of MSCs *in vitro* and attempted to elucidate the underlining regulatory mechanisms involving METTL3 and the PI3K/AKT signaling pathway.

## EXPERIMENTAL PROCEDURES

**Cell isolation and culture**

MSCs were collected from the bone marrow of Sprague-Dawley (SD) rats (male, weighing between 80 and 120 g) as previously described [19]. All SD rats were purchased from Sun Yat-sen University (Guangzhou, China), and the study procedures were approved by the Animal Ethic Committee of Sun Yat-sen University. Briefly, bone marrow in femurs and tibias from the SD rats were flushed using sterile PBS with 1% penicillin/streptomycin (100 U/mL, HyClone, USA). The cell suspension was centrifuged at 1,000 rpm for 5 min. Then, the supernatant was removed and the cell pellet was resuspended in 4 mL of low-glucose Dulbecco’s modified Eagle’s medium (DMEM; GIBCO, USA) containing 10% fetal bovine serum (FBS; GIBCO, USA) and 1% penicillin/streptomycin (100 U/mL, HyClone, USA), before being plated in a 25 cm^2^ flask. When the confluence of adherent cells reached 90%, digestion passage was performed at a dilution of 1 : 2. MSCs from the third-passage were positive for CD44 and CD29 but negative for CD34 [27], making them useable for subsequent experiments.

**ELA treatment**

The ELA containing 32 amino acids (sequence: QR PVNLTMRRKLRKHNCLQRRCMPLHSRVPFP) was synthesized by GL Biochem Shanghai Ltd (China). To investigate the effects of ELA on the enhancement of cell proliferation and migration, the MSCs were treated with 5 µM ELA for 24 h. The MSCs were treated with ELA at 0 to 40 μM for 24 h to investigate the relationship between ELA and METTL3 in MSCs. To investigate the signaling pathway downstream of ELA, 5 µM of ELA was added into the culture medium post-transfection for 24 h.

**Cell transfection**

Small interfering RNA (siRNA) targeting METTL3 (si-METTL3, sequence: CCTACAAGATGACGCACAT), APJ (si-APJ, sequence: GCCTCAGCTTTGACCGATA) and their negative control (NC) were synthesized by RiboBio Co. (Guangzhou, China). Lipofectamine RNAiMax Reagent (Thermo Fisher, USA) was employed for siRNA transfection into the MSCs. Briefly, MSCs were cultured in plates with a penicillin/streptomycin-free medium. The transfection reagent and siRNA (50 nM) were dissolved in DMEM and mixed for 20 min before being dropped onto the culture plate.

**CCK-8 assay**

The Cell Counting Kit-8 (CCK-8; Dojindo Laboratories, Kumamoto, Japan) was used to detect cell proliferation and viability. The cells were seeded onto 96-well plates (3,000 cells per well) and treated accordingly. Afterwards, the cells were incubated with the CCK-8 working solution for 2 h. The optical density (OD) values at a wavelength of 450 nm were evaluated using a microplate reader (Thermo Varioskan LUX, USA).

**EdU staining assay**

The 5-ethynyl-2’-deoxyuridine (EdU) assay kit (Ribobio, Guangzhou, China) was applied to evaluate cell proliferation. The MSCs were seeded in a 96- well plate. After the designated treatment, 50 µ mol/L of EdU was added to the MSC medium and incubated for 2 h. Afterward, the MSCs were fixed with 4% paraformaldehyde and permeabilized with 0.5% Triton-X 100. After rinsing, the MSCs were incubated with the staining reagent (EdU Apollo) for 30 min. Consequently, all cell nuclei were stained with the Hoechest solution (1 : 1 000) and visualized with a confocal laser scanning microscope (ZEISS, Germany).

**Transwell assay**

The Transwell system (8-mm pore, Corning, Beijing, China) was applied to analyze the migration capability of MSCs. Cells were incubated with 0.25% trypsin (Gibco, USA) and resuspended in a serum-deficient (0.5% FBS) medium. A cell suspension containing 6 × 10^4^ cells was added to the upper chamber, whereas a 600 µL medium containing 10% FBS was added into the bottom chamber. After incubation for 8h, the cells on the upper surface were wiped with cotton swabs and the cells which had migrated to the lower surface were fixed with 4% paraformaldehyde for 30 min and then stained with 0.1% crystal violet for 20 min. Subsequently, the membrane was airdried after washing with PBS and 5 fields were randomly examined under the microscope (ZEISS, Germany).

**Measurement of the m_6_A methylation level**

The level of m_6_A modification in MSCs was measured using the EpiQuik m_6_A RNA Methylation Quantification Kit (Epigentek, USA). Briefly, 200 ng of total RNA was bound to the wells using the Binding Solution. Then, Capture Antibody, Detection Antibody, and Enhance Solution were added into the wells. After washing, Development Solution and Stop Solution were used to complete the reaction. Absorbance at 450 nm was evaluated using a microplate reader (Thermo Varioskan LUX, USA).

**Western blot analysis**

MSCs were lysed by a RIPA lysis buffer (Beyotime, China) supplemented with a protease inhibitor and a phosphatase inhibitor (CWBIO, China). After 30 min, the cell lysate was centrifuged at 12,000 rpm for 15 min. The supernatant was collected, and the protein concentration was measured by using the bicinchoninic acid (BCA) protein assay (CWBIO, China). After adding the loading buffer, the protein samples were heated for 10 min at 100°C. Equal-sized samples were separated by 10% SDS-PAGE and then transferred onto a polyvinylidene fluoride (PVDF) membrane (0.45 µm, Millipore, USA). Then, after incubation with 5% skim milk for 1 h, the membranes were incubated with the primary antibody at 4°C overnight: GAPDH (1 : 1000; # 2118; Cell Signaling Technology, USA), APJ (1 : 1000; # bs-2430R; Bioss, CHINA), phospho-Akt (Ser473) (1 : 2000; # 4060; Cell Signaling Technology, USA), AKT (1 : 1000; # 4691; Cell Signaling Technology, USA), METTL3 (1 : 1000; # 96391; Cell Signaling Technology, USA). After being washed with 1× TBST (3 times, per 5 min), the membranes were incubated with the secondary antibody (goat anti-rabbit IgG coupled with HRP, 1 : 2000, Cell Signaling Technology, USA) at room temperature for 1 h. About 1× TBST (3 times, per 5 min) was used to rinse the membranes, and chemiluminescence reagents were used to identify the bands by the ChemiDoc™ Touch Imaging System (Bio-Rad, USA).

**Statistical analysis**

The data were expressed in the form of mean ± SD. All experiments were performed independently at least three times. One-way analysis of variance (ANOVA) was used for multigroup comparisons, and the Tukey’s post-hoc test was used for comparisons between two independent groups. The significance of the difference between the two groups was evaluated by the Student’s *t*- test, with *P* < 0.05 being considered as statistically significant.

## RESULTS AND DISCUSSION

**Results**

**Fig. 1:**
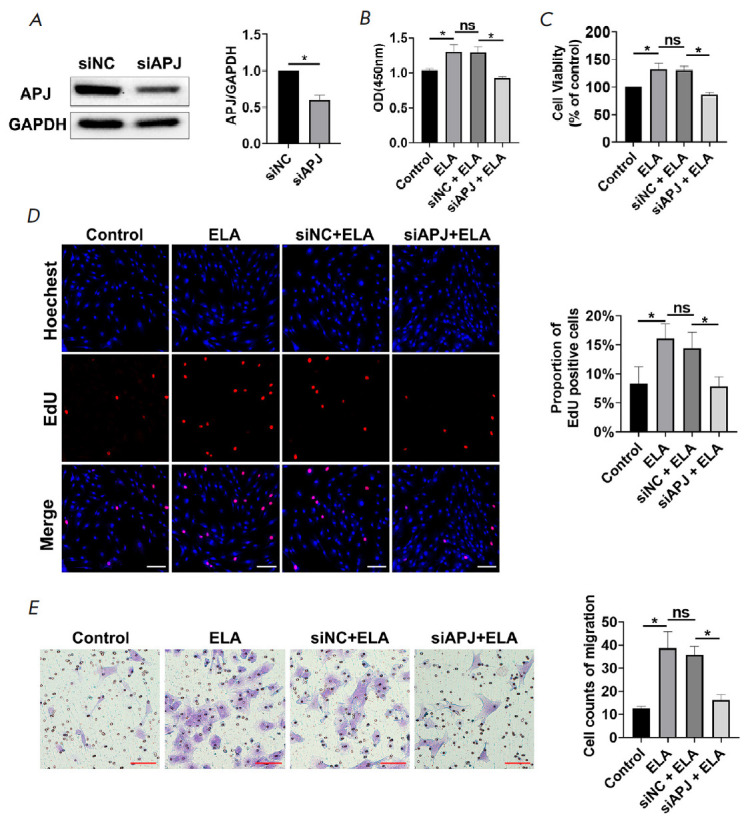
ELA promotes the proliferation and migration of MSCs in an APJ-dependent manner. (*A*) – Knockdown efficiency of siAPJ evaluated by Western blot. (*B*, *C*) – CCK-8 assay results on MSC proliferation capacity and viability of various experimental groups. (*D*) – The representative images and quantification of EdU assay are shown. Scale bar 100 μm. (*E*) – Transwell assay was performed to detect MSC migration. Scale bar 100 μm. *P < 0.05; ns – no significance; siAPJ – APJ knockdown; siNC – negative control

*ELA promotes the proliferation and migration of MSCs in an APJ-dependent manner.* The MSCs were treated with 5 μM of ELA to investigate the effect of ELA on MSC proliferation and migration. The CCK8 data revealed ELA-treated MSCs to show improved proliferation and viability compared with the control group (*Fig. 1 B,C*), while data from the EdU assay indicated the percentage of EdU-positive cells to be markedly increased in the ELA group (*Fig. 1D*). In line with the results of the CCK8 and EdU assays, the migration ability of MSCs was increased after treatment with ELA (*Fig. 1E*). These results appeared to show the ability of ELA to promote MSC proliferation and migration. APJ is a known receptor for ELA. To verify the possible regulatory role of ELA through APJ, the expression of APJ in MSCs was downregulated with small interfering RNAs. The results in *Fig. 1A* demonstrate the successful knockdown of APJ in the siAPJ group. It was discovered that the siAPJ + ELA group exhibited significantly decreased proliferation ability, cell viability, as well as cell migration when compared with the siNC + ELA group (*Fig. 1*). The results between the siNC + ELA and ELA groups showed no significant difference. In summary, it appears that ELA may promote the proliferation and migration capability of MSCs in an APJ-dependent manner.

**Fig. 2:**
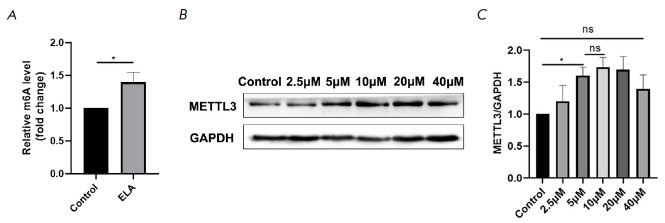
ELA upregulates the m_6_A level and the protein level of METTL3 in MSCs. (*A*) – ELA upregulated the level of m_6_A RNA methylation in MSCs. (*B*, *C*) – ELA increased the protein expression of METTL3 in a concentration-dependent manner ranging from 5–20 μM. *P < 0.05; ns – no significance

*ELA upregulated the m_6_A level and the protein level of METTL3 in MSCs. *Recent studies have shown that m_6_A plays an important role in various biological functions of cells, such as proliferation and migration. Therefore, to further explore the mechanism by which ELA promotes the proliferation and migration ability of MSCs, we sought to establish the m_6_A level in MSCs. The results showed that, compared with the control group, the m_6_A level in ELA-treated MSCs was significantly upregulated (*Fig. 2A*).

As the main component of the m_6_A methyltransferase complex (MTC), METTL3 has a definitve influence on the regulation of stem cell function. Therefore, the expression of METTL3 was measured under increasing concentrations (0−40 μM) of ELA. As shown in *Fig. 2 B,C*, the protein level of METTL3 increased in a concentration-dependent fashion within a certain concentration gradient (5−20 μM). No significant difference was observed at 40 μM compared with the control group. These results indicated that ELA could upregulate the expression of METTL3. Based on these results, it appeared reasonable suspect that ELA might affect the proliferation and migration of MSCs by regulating the protein expression of METTL3.

**Fig. 3:**
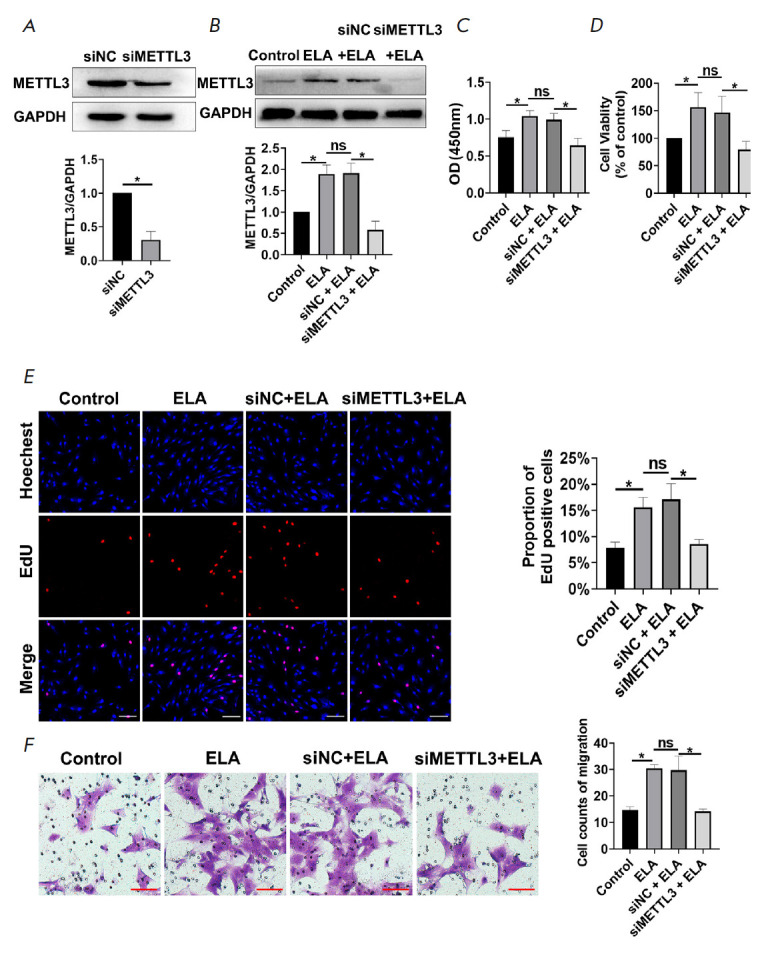
ELA promotes MSC proliferation and migration by upregulating METTL3. (*A*) – METTL3 knockdown was successfully performed using small interfering RNA. (*B*) – METTL3 knockdown attenuated the upregulation of METTL3 caused by ELA at the protein level. (*C*, *D*) – CCK-8 assay results demonstrate that METTL3 knockdown reversed the synergistic effects of ELAenhanced MSC proliferation and viability. (*E*) – EdU assay shows that METTL3 knockdown decreases the percentage of EdU-positive MSCs that was increased by ELA. Scale bar 100 μm. (*F*) – Transwell assay results show the inhibitory effects of METTL3 knockdown on cell migration was increased by ELA. Scale bar 100 μ m. *P < 0.05; ns – no significance; siMETTL3 – METTL3 knockdown; siNC – negative control

*ELA promotes MSC proliferation and migration by regulating the expression level of METTL3. *To validate the hypothesis that ELA may promote MSC proliferation and migration by regulating the expression of METTL3, METTL3 knockdown was performed in MSCs and the protein level was examined by Western blot (*Fig. 3A*). The analysis showed ELA to upregulate the METTL3 protein level in MSCs, and this effect was reversed by METTL3 knockdown (*Fig. 3B*).

In *Fig. 3 C,D*, cell proliferation and viability were observed to increase in the presence of ELA compared with the control group, while it was significantly decreased in the siMETTL3 + ELA group compared with the siNC + ELA group. Moreover, compared with the control group, the ratio of EdUpositive MSCs increased upon ELA treatment but decreased in the METTL3 knockdown group (*Fig. 3E*). Consistent with the results for proliferation, METTL3 knockdown also appeared to decrease cell migration when compared with the ELA group and the siNC + ELA group (*Fig. 3F*). In addition, no distinct difference was observed between the ELA group and siNC + ELA group (*Fig. 3*). Therefore, it appears reasonable to infer that METTL3 deficiency may block the promotion effect of ELA on MSC proliferation and migration.

**Fig. 4:**
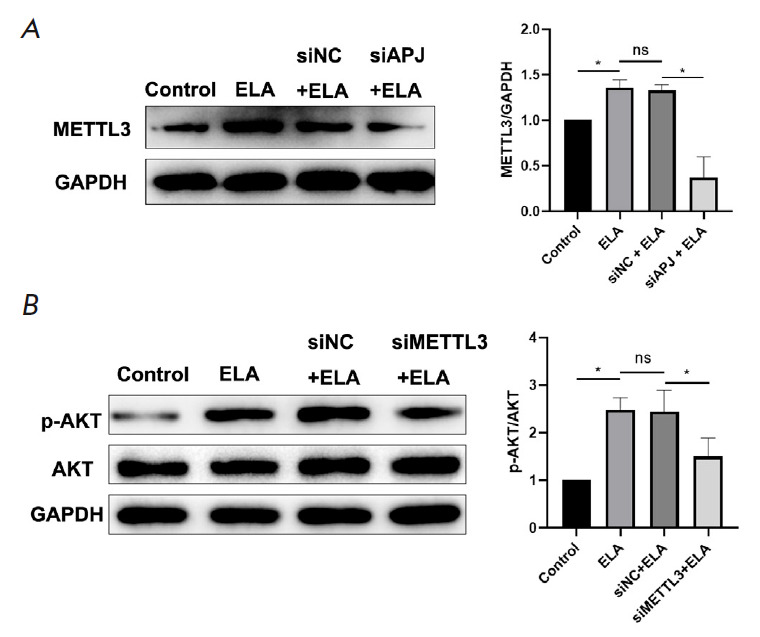
ELA upregulates the expression of p-AKT by enhancing APJ-associated METTL3 upregulation. (*A*) – Western blot results show that APJ knockdown reversed the upregulation of METTL3 caused by ELA. (*B*) – The downregulation of METTL3 inhibited the increasing ratio of p-AKT/AKT caused by ELA. *P < 0.05; ns – no significance; p-AKT – phosphorylated AKT; siAPJ – APJ knockdown; siMETTL3 – METTL3 knockdown; siNC – negative control

*ELA/APJ activates the PI3K/AKT pathway by upregulating the expression of METTL3. *To explore the regulatory mechanism behind the effects of ELA on the MSC proliferation and migration abilities, the expression level of METTL3 and the key kinase of the PI3K/AKT pathway were further assessed. As shown in *Fig. 4A*, METTL3 was observed to be markedly upregulated in the ELA group compared with the control group, but this effect ceased upon APJ knockdown, indicating the ability of ELA to upregulate METTL3 expression through the APJ receptor. Additionally, previous studies confirmed that ELA could regulate the PI3K/AKT pathway in MSCs [19], and that this pathway is regulated by METTL3 [26]. Therefore, further experiments were carried out to check AKT phosphorylation, with Western blot results confirming increased levels of p-AKT after treatment with ELA. However, METTL3 knockdown reversed this pattern (*Fig. 4B*), suggesting that ELA had activated the APJ receptor, which promotes the proliferation and migration capacity of MSCs, via the METTL3/PI3K/AKT pathway.

**Discussion**

In this study, the effects and underlying mechanisms of ELA vis-a-vis MSCs were evaluated. Our results revealed that ELA could promote the proliferation, viability, and migration of MSCs *in vitro *in an APJFig dependent manner. In addition, ELA was shown to upregulate the m_6_A level and the protein level of METTL3 in MSCs and to activate the PI3K/AKT pathway. METTL3 knockdown not only reversed the effect of ELA on cellular proliferation and migration, but also inhibited the synergistic effect of ELA on p-AKT, suggesting the activation of the METTL3/ PI3K/AKT axis as the underlying mechanism. At present, no research has reported any relationship between ELA and m_6_A, but the results from this study have confirmed that the ELA-APJ signaling pathway may have synergistic effects on the proliferation and migration of MSCs by activating the METTL3/PI3K/AKT axis. This finding provides new strategies for improving the expansion and migration abilities of MSCs *in vitro *and partially sheds light on the potential mechanism of ELA in terms of its promoting effects on MSCs.

The results of this study show that the administration of exogenous ELA can lead to improved proliferation, viability, and migration ability for MSCs. Growing evidence points to the critical role of ELA in the biological functions of the cell [28]. Ho and colleagues confirmed that ELA could improve the proliferation ability of hESCs via the PI3K/Akt pathway [14]. The PI3K/Akt signaling pathway regulates multiple cellular processes of MSCs such as proliferation [16] and migration [17], implying that ELA exerts these effects on MSCs through the PI3K/Akt signaling pathway. Consistent with our results, previous studies have demonstrated that the ELA-APJ signaling pathway stimulates cell motility [12] and influences angioblast migration [13] during vasculogenesis. Meanwhile, the activation of APJ has been reported to promote cellular proliferation and migration [29]. Thus, the effect of ELA in MSCs is most likely mediated through APJ. Ho and colleagues also suggested that ELA can function through an alternate receptor in hESCs [14]. However, it was observed in this study that APJ knockdown reversed the proliferation and migration abilities induced by ELA in MSCs, making APJ the key receptor of ELA in MSCs. Based on these results, ELA should be considered as a complement for the *in vitro *expansion of MSCs, besides its role as improving MSC migration.

At present, little is known about the molecular mechanisms of ELA in various biological functions. This research confirms that the expression level of the METTL3 protein is considerably upregulated by ELA in a concentration-dependent manner within 5−20 μM. A significant difference was observed from the concentration of 5 μM onward, which was consistent with the concentration used in the cell proliferation experiment. This concentration was thus used in all subsequent experiments. N6-Methyladenosine (m_6_A), which accounts for the most prevalent RNA internal modification in eukaryotes, plays a critical role in various bioprocesses and diseases, such as stem cell self-renewal [30], differentiation [31], and tumorigenesis [32]. METTL3 is the active component in the m_6_A methyltransferase complex that has been confirmed to be implicated in biological functions such as cell proliferation and migration [23]. A previous study demonstrated the oncogenic effects of METTL3 in breast cancer [33]. Furthermore, Tian et al. found that Mettl3 knockdown suppressed the activation of the PI3K/AKT pathway during the process of MSC osteogenic differentiation in [26], and this pathway has been confirmed to be initiated by ELA [19]. Therefore, based on these results, it appeared legitimate to speculate that ELA affects MSC proliferation and migration by regulating the expression of METTL3. The protein level of METTL3 was then downregulated, and it was noticed that the promoting effects of ELA on the proliferation and migration of MSCs were significantly attenuated upon METTL3 knockdown. It is thus concluded that ELA futhers the proliferation and migration abilities of MSCs by upregulating the expression of METTL3. This is the first report of the regulatory effect of ELA on the expression of METTL3.

When the mechanism by which the ELA-induced function changes in MSCs was explored, the phosphorylation level of AKT was observed to be upregulated in ELA-treated MSCs. Activation of the PI3K/AKT pathway could enhance cell-cycle progression through the G1/S phase to promote cell proliferation [34], whereas increased expression of MMP-2 and MMP-9 appeared to stimulate cell migration [35]. Additionally, fate determination of bone marrow MSCs could be regulated by METTL3 via PI3KAkt signaling [26], indicating that ELA might activate the PI3K-Akt signaling by upregulating the expression of METTL3. Therefore, METTL3 expression was downregulated and METTL3 knockdown was found to inhibit the activation of the PI3K/AKT signaling pathway caused by ELA. Based on the results of this study, it can be concluded that ELA/APJ signaling affects proliferation and migration via the METTL3/ PI3K/AKT pathway. However, it is worth noting that the downstream target genes of METTL3 have yet to be explored in this study. Therefore, future research should focus on identifying the genes that are regulated by METTL3 to fully clarify the mechanism of ELA action on MSCs, and whether METTL3 functions in an m_6_A-dependent manner.

## CONCLUSION

Over all, this study provides evidence that support the role of ELA/APJ signaling in promoting the proliferation and migration of MSCs, which may be a result of the activation of the METTL3/PI3K/AKT pathway.

## Abbreviations

MSCs: mesenchymal stem cells
ELA: ELABELA
APJ: apelin receptor
PI3K: phosphatidylinositol 3 kinase
METTL3: Methyltransferase-like 3
m6A: N6-methyladenosine
hESCs: human embryonic stem cells
MTC: methyltransferase complex
HSPCs: human hematopoietic stem/ progenitor cells
siRNA: small interfering RNA
p-Akt: phosphorylated Akt
